# The complete chloroplast genome sequence and phylogenetic analysis of *Amorphophallus gigas*

**DOI:** 10.1080/23802359.2026.2728751

**Published:** 2026-09-10

**Authors:** Wei Wang, Kai Chen, Jiamin Mu, Ying Qi, Min Yang, Meiwei Zhao, Penghua Gao, Feiyan Huang, Lixiang Li, Lifang Li, Lei Yu

**Affiliations:** Yunnan Key Laboratory of Konjac Biology, College of Agronomy, Yunnan International Joint Laboratory of Konjac Resources Conservation and Utilization, Kunming University, Kunming, China

**Keywords:** Complete chloroplast genome, *amorphophallus gigas*, phylogenomic, araceae

## Abstract

We sequenced the complete chloroplast genome of* Amorphophallus gigas*, a perennial monocotyledonous herb in Araceae*,* using HiFi technology*.* The genome is 173,034 bp in length with a GC content of 34.96%*.* It exhibits a typical quadripartite structure: a large single-copy (LSC) region of 95,283 bp, a small single-copy (SSC) region of 15,675 bp, and a pair of inverted repeat (IR) regions of 31,038 bp each. It encodes 130 genes (85 protein-coding, 37 tRNA, 8 rRNA). Phylogenetic analysis revealed that* A. gigas* is closely related* to A. titanum*, forming a distinct clade*.* This study provides valuable genomic resources for understanding the evolution of* Amorphophallus* and Araceae.

## Introduction

The genus *Amorphophallus* (family Araceae) comprises perennial herbs with a wide distribution, spanning from tropical Africa through subtropical Asia to the tropical Western Pacific and Northeastern Australia. This genus includes approximately 230 species globally, of which more than 20 are found in China, establishing the country as one of its diversity centers (Srzednicki and Borompichaichartkul 2020). Some *Amorphophallus* species are valued for their corms rich in konjac glucomannan (KGM), demonstrating broad application potential across various high-technology fields including food, pharmaceuticals, chemical industry, and agriculture (Ye et al. 2022; Jin et al. 2024). *A. gigas* Teijsm. & Binn. 1862 produces tubers with potential for medicinal and food use (Esther et al. 2024).

*A. gigas* is the second largest species in the genus in terms of overall structure, following *Amorphophallus titanum*, yet it possesses the tallest inflorescence among all known *Amorphophallus* species (Ittenbach 1998). Morphologically, *A. gigas* is characterized as a monophyllous herb featuring a dark green petiole adorned with light green spots of varying sizes. The petiole measures 3–4 m in length and 11–20 cm in diameter, with a crown spread of up to 4 m, while the stem can attain a length of 4 m, contributing to its notably tall vegetative stature (Rugayah et al. 2017; Liu et al. 2019). Despite its significant resource potential, the survival of *A. gigas* is precarious. Its limited distribution range is threatened by two primary factors: habitat loss due to land conversion and over-exploitation through tuber harvesting (Ridahati et al. 2025). Chloroplast genomes are a powerful tool for plant phylogenetics, species identification, and genetic resource assessment, owing to their conserved structure, uniparental inheritance, and high copy number, which facilitate the efficient detection of genetic differences and evolutionary relationships (Li et al. 2022). Despite this utility, prior research on *A. gigas* has focused predominantly on ecological aspects, such as population reproduction and environmental interactions, with its chloroplast characteristics remaining unexplored. To address this gap, we present the first complete chloroplast genome of *A. gigas*, detailing its sequencing, assembly, and annotation. We further analyze its sequence characteristics, gene composition, and structural features to preliminarily clarify its phylogenetic position within the genus.

## Materials and methods

### Plant materials, DNA extraction, and sequencing

**Figure 1.**
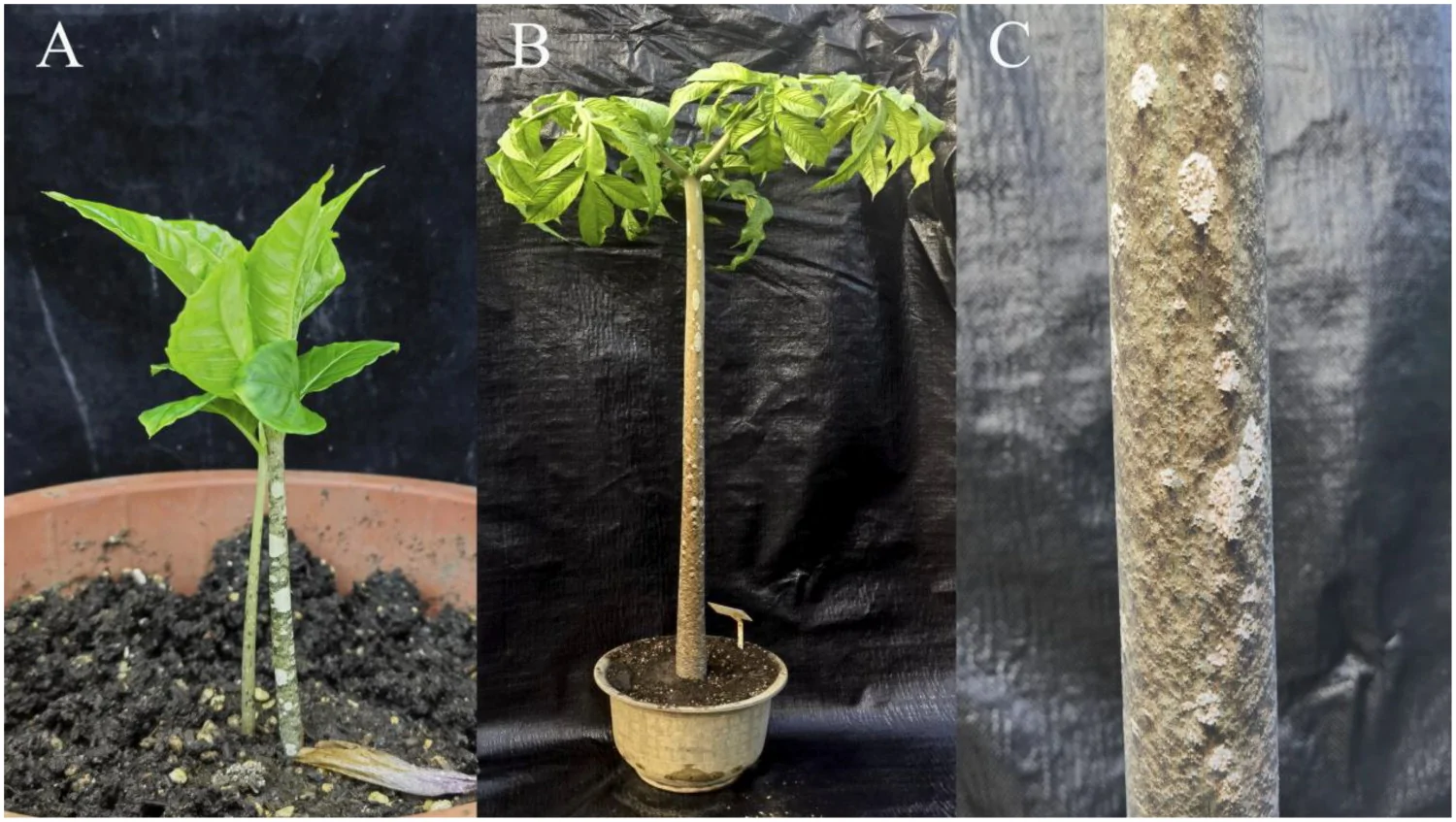
Morphological characteristics of *Amorphophallus gigas*. (A) Annual plant, displaying a single leaf with a broad-ovate to cordate lamina, entire margin, and thick texture. (B) Perennial plant, exhibiting paired, highly dissected compound leaves with oblong to obovate leaflets and undulate margins. (C) Detail of the petiole of a perennial plant. The petiole is dark greenish-brown, densely covered with light-colored patches, and has a dense surface. Photographs were taken by Yu Lei and Wang Wei in the greenhouse of Kunming University.

Young and healthy leaves of *A. gigas* were collected from the Yunnan International Joint Laboratory for Conservation and Utilization of Konjac Resources, Kunming University, Yunnan Province, China (Figure 1, longitude 102°47′46″ E and latitude 24°58′26″N). A voucher specimen was deposited in the Herbarium of the Yunnan Urban Agricultural Engineering and Technological Research Center, Kunming University (voucher number: XJMY20250616, Wei Wang, 3219297536@qq.com). The mature, healthy leaves, free from pests and diseases, were collected, rinsed with sterilized water, blotted dry, immediately flash‑frozen in liquid nitrogen, and stored at ‑80 °C until further analysis. Genomic DNA was extracted using the DNeasy Plant Mini Kit (Qiagen, Hilden, Germany). The purity and concentration of the extracted genomic DNA were assessed using a NanoDrop 1000 spectrophotometer (Thermo Fisher Scientific) and a Qubit fluorometer (Thermo Fisher Scientific). Subsequently, a 15 kb library was constructed following the manufacturer’s instructions of the SMRTbell Express Template Prep Kit 2.0 (Pacific Biosciences, USA) and sequenced on the PacBio Revio platform (Pacific Biosciences, USA).

### Sequence assembly and annotation

Raw sequencing data were processed with the CCS algorithm (v6.0.0, parameters: –minPasses 3 –min predicted accuracy 0.99 –max Length 21,000) to generate high-quality HiFi reads. These HiFi reads were then aligned to a reference chloroplast genome (NC_056329.1, *A. titanum*) using minimap2 (v2.15-r905), producing a PAF (Pairwise mApping Format) file. Based on this alignment, successfully mapped reads were retained, and those with a coverage depth below 50× were filtered out. The filtered reads were then *de novo* assembled into the complete chloroplast genome using Flye v2.6 (Kolmogorov et al. 2020), representing a *de novo* assembly strategy. The resulting assembly graph was visualized using Bandage (v0.8.1) to verify structural completeness and accuracy. The complete chloroplast genome sequence was annotated using the PGA (Plastid Genome Annotator) software, followed by manual curation. Finally, a circular genome map was generated using the OGDRAW program (Greiner et al. 2019).

### Phylogenetic analysis

To determine the phylogenetic placement of *A. gigas*, we retrieved the complete chloroplast genome sequences of 18 related species from the NCBI database. The dataset comprised 6 species from the genus *Amorphophallus* and 11 species from other genera within the family Araceae. *Iris missouriensis* (Iridaceae) was used as the outgroup. Multiple sequence alignment of the complete chloroplast genomes from all 19 taxa (including *A. gigas* sequenced in this study) was performed using MAFFT v7, followed by manual refinement with BioEdit (Katoh and Standley 2013). Based on the resulting alignment, the optimal nucleotide substitution model was determined using ModelFinder. Subsequently, a maximum likelihood (ML) phylogenetic tree was constructed with IQ-TREE v1.6.12 under the best-fit model GTR+F + I + G4, with branch support measured from 1000 bootstrap replicates (Nguyen et al. 2015; Kozlov et al. 2019). The final tree was visualized using FigTree v1.4.3 (Yin et al. 2021).

## Results

**Figure 2.**
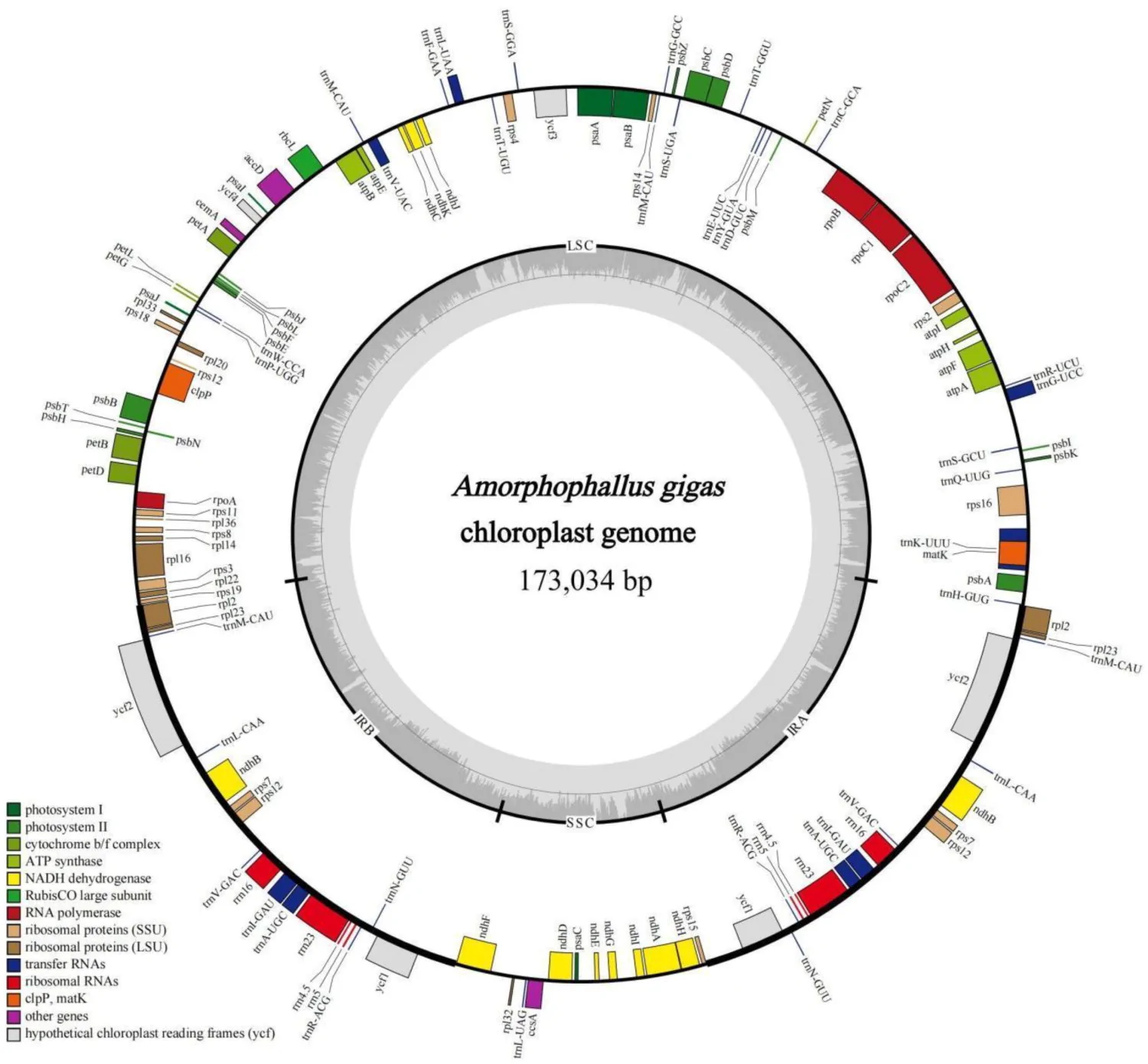
Circular map of the *A. gigas* chloroplast genome. Genes situated inside the circle are transcribed in the counterclockwise direction, whereas those outside are transcribed clockwise. The functional categories of genes are indicated by distinct colors. The inner gray histogram represents the GC content, with the Central gray line indicating the 50% threshold.

**Figure 3.**
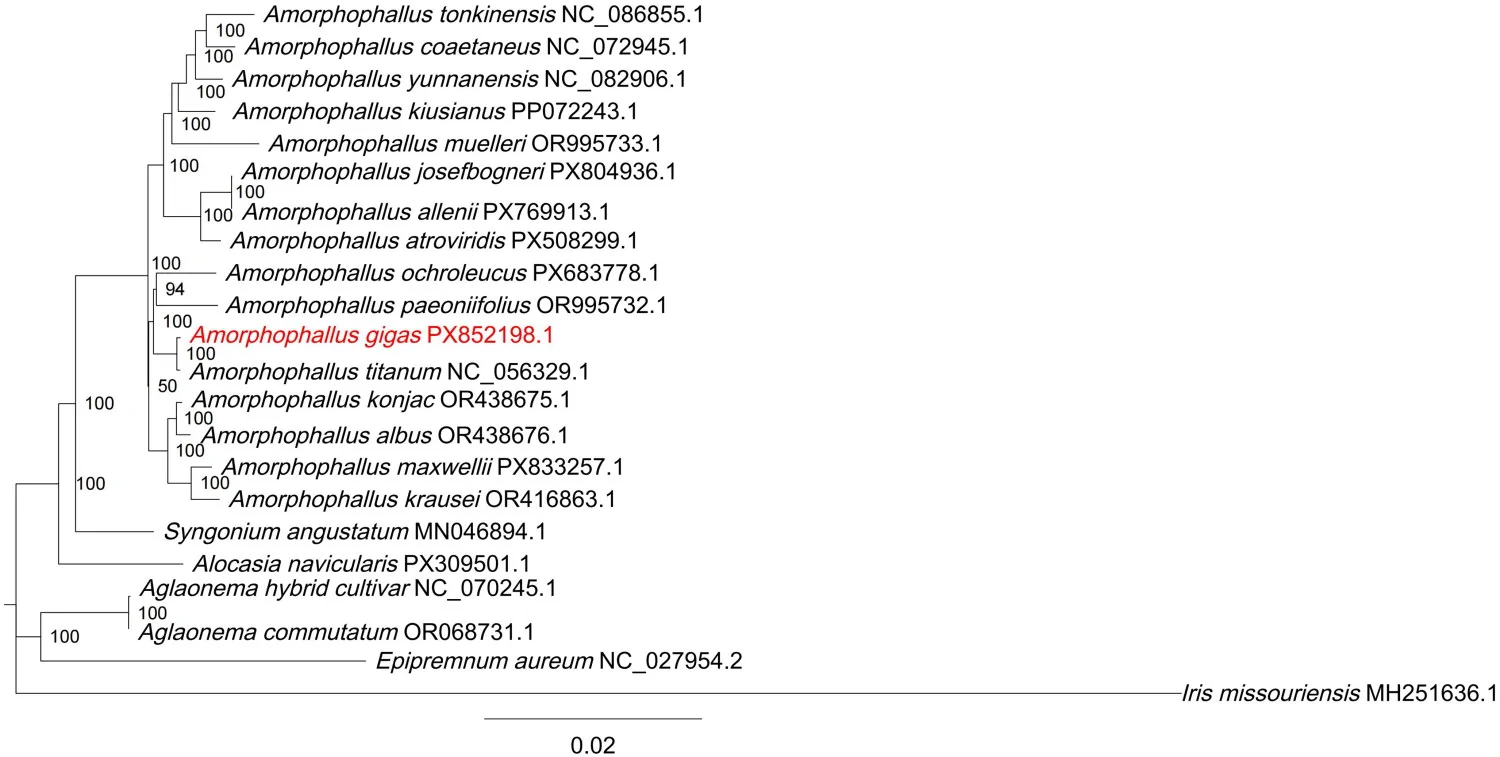
Phylogenomic tree of *A. gigas* and related species. The tree was constructed using the maximum likelihood method based on complete chloroplast genome sequences, with *iris missouriensis* serving as the outgroup. The optimal substitution model, selected according to the bayesian information criterion, was GTR+F + I + G4. The scale bar represents the number of substitutions at each site. Numbers at nodes represent bootstrap support values based on 1000 replicates. The sequences used are as follows: *Amorphophallus tonkinensis* NC_086855.1 (Yin et al. 2024), *A. coaetaneus* NC_072945.1 (Gao et al. 2023), *A. yunnanensis* NC_082906.1 (Yin and Gao 2023a), *A. kiusianus* PP072243.1 (Gao and Yin 2024), *A. muelleri* OR995733.1 (Li et al. 2024b), *A. josefbogneri* PX804936.1 (Shi et al. 2026a), *A. allenii* PX769913.1 (Shi et al. 2026b), *A. atroviridis* PX508299.1 (Shi et al. 2026c), *A. ochroleucus* PX683778.1 (Shi et al. 2026d), *A. paeoniifolius* OR995732.1 (Li et al. 2024a), *A. titanum* NC_056329.1 (Abdullah et al. 2021), *A. konjac* OR438675.1 (Li et al. 2024c), *A. albus* OR438676.1 (Li et al. 2024b), *A. maxwellii* PX833257.1 (Shi et al. 2026e), *A. krausei* OR416863.1 (Yin and Gao 2023b), *syngonium angustatum* MN046894.1 (Henriquez et al. 2020a), *alocasia navicularis* PX309501.1 (unpublished), *aglaonema hybrid cultivar* NC_070245.1, *A. commutatum* OR068731.1 (unpublished), *epipremnum aureum* NC_027954.2 (unpublished) , *iris missouriensis* MH251636.1 (unpublished).

The complete chloroplast genome of *A. gigas* is 173,034 bp in length, with an average GC content of 34.96% and an average sequencing depth of 638× (Figure S1). Consistent with previously reported chloroplast genomes of other plants, it exhibits a typical quadripartite structure. It is composed of a large single-copy (LSC) region of 95,283 bp, a small single-copy (SSC) region of 15,675 bp, and two inverted repeat (IRA and IRB) regions, each measuring 31,038 bp (Table S1, Figure 2). The assembled and annotated chloroplast genome sequence has been deposited in the GenBank database under accession number PX852198. This genome encodes 130 genes (111 unique genes), including 85 protein-coding genes, 8 rRNAs, and 37 tRNAs (Table S1). Among these, 7 tRNA genes (*trn*H-GUG, *trn*K-UUU, *trn*G-UCC, *trn*L-UAA, *trn*V-UAC, *trn*I-GAU, and *trn*A-UGC) and 9 protein-coding genes (*rps1*6, *atp*F, *rpo*C1, *pet*B, *pet*D, *rpl*16, *rpl*2, *ndh*B, and *ndh*A) contain a single intron, while 3 genes (*rps*12, *clp*P, and *ycf*3) contain two introns (Table S2 and Figure S2), and one trans-splicing gene (*rps*12) was observed (Figure S3). Most genes (111) are present in a single-copy. 19 genes located within the inverted repeat (IR) regions are duplicated, encompassing all 4 rRNA genes, 8 tRNA genes, and 7 protein-coding genes (Table S2). To elucidate the phylogenetic relationships within Araceae, a phylogenetic tree was constructed based oncomplete chloroplast genome sequences. Maximum Likelihood (ML) analysis revealed that all species of the genus *Amorphophallus* formed a monophyletic clade with high statistical values. Within this clade, *A. gigas* was more closely associated with *A. titanum* (NC_056329). Furthermore, the genera *Amorphophallus* and *Syngonium* were identified as sister taxa (Figure 3).

## Discussion and conclusion

In this study, we present the first complete chloroplast genome sequence of *A. gigas*, which was assembled using PacBio HiFi technology. The genome has a total length of 173,034 bp and contains 130 genes. Consistent with the typical quadripartite structure of most angiosperm chloroplast genomes, it is organized into two inverted repeat (IRa and IRb) regions that separate a large single-copy (LSC) and a small single-copy (SSC) region. The overall GC content was 34.96%, comparable to values reported for other *Amorphophallus* species (Huan et al. 2019; Abdullah et al. 2021; Li et al. 2024c). The chloroplast genome of *A. gigas* encodes 130 genes (111 unique genes), a count that falls within the genus-specific range of 128 to 132 genes. This aligns with documented gene numbers in other *Amorphophallus* species: 128 genes in *A. yunnanensis* (Yin and Gao 2023a), 130 in *A. paeoniifolius* (Li et al. 2024a), 131 in *A. konjac* (Huan et al. 2019), and 132 in both *A. krausei* (Yin and Gao 2023a) and *A. kachinensis* (Gao and Yin 2024). Maximum Likelihood (ML) phylogenetic analysis confirmed that species of the genus *Amorphophallus* form a monophyletic clade with high statistical support, a finding consistent with previous studies (Yin and Gao 2023a; Li et al. 2024b). Within this clade, *A. gigas* shows a closer phylogenetic affinity to *A. titanum* than to other congeners. This study not only enriches the genomic resources available for *A. gigas* but also provides a foundation for further investigation into the genetic diversity, evolutionary history, and phylogenetic relationships within the genus *Amorphophallus*.

## Supplementary Material

Supplemental material.doc

Table S2 Genes in Amorphophallus gigas chloroplast genome.doc

Table S1 General features of the Amorphophallus gigas chloroplast genome.doc

## Data Availability

The assembled chloroplast genome sequence data that support the findings of this study are openly available in GenBank of NCBI (https://www. ncbi.nlm.nih.gov/) under the accession number of PX852198. The associated BioProject, SRA, and Bio-Sample numbers are PRJNA1397504, SRR36665509, and SAMN54432800, respectively.
