# Supplementary material for: The complete chloroplast genome sequence and phylogenetic analysis of Amorphophallus gigas
Source: Mitochondrial DNA B Resour. 2026 Sep 10;11(10):1146–50. doi: 10.1080/23802359.2026.2728751 (PMC13564156; doi:10.1080/23802359.2026.2728751)
Supplement: Supplementary file 1 [file TMDN_A_2728751_SM2049.doc]

Table S1. General features of the *Amorphophallus gigas* chloroplast genome

| Nomenclature | Value |
| --- | --- |
| Total length | 173034 bp |
| LSC | 95283 bp |
| SSC | 15675 bp |
| IR | 31038 bp |
| GC% | 34.96% |
| Total | 130 |
| Protein coding genes | 85 |
| rRNA | 8 |
| tRNA | 37 |
| JLB | 95284 |
| JSB | 126321 |
| JSA | 141997 |
| JLA | 173034 |
