# Supplementary material for: The complete chloroplast genome sequence and phylogenetic analysis of Amorphophallus gigas
Source: Mitochondrial DNA B Resour. 2026 Sep 10;11(10):1146–50. doi: 10.1080/23802359.2026.2728751 (PMC13564156; doi:10.1080/23802359.2026.2728751)
Supplement: Supplementary file 3 [file TMDN_A_2728751_SM2051.doc]

Supplemental material

Figure S1. Coverage depth figure of the *Amorphophallus gigas* chloroplast genome. The horizontal coordinate is the base of the chloroplast genome and the vertical coordinate is the depth of sequencing corresponding to that base.

Figure S2. The map of the cis-splicing genes in the *A. gigas* chloroplast genome.

Figure S3. The map of the trans-splicing gene *rps*12 in the *A.gigas* chloroplast genome.
